# Reproducibility of EEG functional connectivity in Alzheimer’s disease

**DOI:** 10.1186/s13195-020-00632-3

**Published:** 2020-06-03

**Authors:** Casper T. Briels, Deborah N. Schoonhoven, Cornelis J. Stam, Hanneke de Waal, Philip Scheltens, Alida A. Gouw

**Affiliations:** 1 Alzheimer Center Amsterdam, Department of Neurology, Amsterdam Neuroscience, Vrije Universiteit Amsterdam, Amsterdam UMC, Amsterdam, the Netherlands; 2 Department of Clinical Neurophysiology, Amsterdam Neuroscience, Vrije Universiteit Amsterdam, Amsterdam UMC, Amsterdam, the Netherlands

**Keywords:** Alzheimer’s disease, EEG, Functional connectivity, Reproducibility

## Abstract

**Background:**

Although numerous electroencephalogram (EEG) studies have described differences in functional connectivity in Alzheimer’s disease (AD) compared to healthy subjects, there is no general consensus on the methodology of estimating functional connectivity in AD. Inconsistent results are reported due to multiple methodological factors such as diagnostic criteria, small sample sizes and the use of functional connectivity measures sensitive to volume conduction. We aimed to investigate the reproducibility of the disease-associated effects described by commonly used functional connectivity measures with respect to the amyloid, tau and neurodegeneration (A/T/N) criteria.

**Methods:**

Eyes-closed task-free 21-channel EEG was used from patients with probable AD and subjective cognitive decline (SCD), to form two cohorts. Artefact-free epochs were visually selected and several functional connectivity measures (AEC(-c), coherence, imaginary coherence, PLV, PLI, wPLI) were estimated in five frequency bands. Functional connectivity was compared between diagnoses using AN(C)OVA models correcting for sex, age and, additionally, relative power of the frequency band. Another model predicted the Mini-Mental State Exam (MMSE) score of AD patients by functional connectivity estimates. The analysis was repeated in a subpopulation fulfilling the A/T/N criteria, after correction for influencing factors. The analyses were repeated in the second cohort.

**Results:**

Two large cohorts were formed (SCD/AD; *n* = 197/214 and *n* = 202/196). Reproducible effects were found for the AEC-c in the alpha and beta frequency bands (*p* = 6.20 × 10^−7^, Cohen’s *d* = − 0.53; *p* = 5.78 × 10^−4^, *d* = − 0.37) and PLI and wPLI in the theta band (*p* = 3.81 × 10^−8^, *d* = 0.59; *p* = 1.62 × 10^−8^, *d* = 0.60, respectively). Only effects of the AEC-c remained significant after statistical correction for the relative power of the selected bandwidth. In addition, alpha band AEC-c correlated with disease severity represented by MMSE score.

**Conclusion:**

The choice of functional connectivity measure and frequency band can have a large impact on the outcome of EEG studies in AD. Our results indicate that in the alpha and beta frequency bands, the effects measured by the AEC-c are reproducible and the most valid in terms of influencing factors, correlation with disease severity and preferable properties such as correction for volume conduction. Phase-based measures with correction for volume conduction, such as the PLI, showed reproducible effects in the theta frequency band.

## Introduction

Alzheimer’s disease (AD) is the most common cause of dementia, and it is expected that 131.5 million people will be living with dementia in 2050 [1]. Electroencephalography (EEG) has been used to understand underlying mechanisms and support the diagnosis of AD [2, 3], but also to observe treatment effects [4]. EEG is a relatively easy, cheap and widely available method and provides functional data with a high temporal resolution which makes it an interesting modality to investigate the disease.

Quantitative resting-state EEG data analyses can roughly be divided into three modalities: spectral decomposition, functional connectivity and network-based analyses. Each of these modalities has shown profound changes in AD compared to healthy subjects [2, 5, 6]. Spectral changes in AD are characterized by a general slowing of the dominant oscillatory brain rhythms. Typically, there is an increase in relative theta and delta power and a decrease in relative alpha and beta power [2, 7]. The analyses of spectral changes are very straightforward and require no more than, for example, a fast Fourier transformation [8] of the oscillatory data. As a result, there is a broad consensus concerning the typical spectral changes in AD. Functional connectivity and network analyses on the other hand require more complex calculations and are subject to choices in the use of different properties of the EEG signal [9]. A broad and general consensus about changes in functional connectivity or brain networks is therefore more complicated.

Various studies have reported changes in functional connectivity in AD. In general, AD is considered to be a disconnecting syndrome [10, 11], showing a lower functional connectivity in patients with dementia due to AD compared to healthy controls [3]. This conclusion is, however, not based on consistent methodology. Many studies suffered from small study populations and poor definition of study populations without using clinical [12] or research criteria [13]. But most importantly, although recommendations have been published [9, 14, 15], there is an absence of a consensus on the methodology of estimating functional connectivity. It is, therefore, not surprising that different and sometimes conflicting results have been reported between measures of functional connectivity in AD. Reproducibility issues like these have recently gained more attention throughout the different fields of science [16]. As scientists are more eager to present new results or discoveries, there is limited motivation to reproduce and publish previously reported findings. Previous results could have been influenced by low statistical power, ‘P-hacking’ or publication bias, and it has been estimated that 85% of biomedical research efforts are not reproducible [17]. This methodological problem is often referred to as the ‘reproducibility crisis’. A lack of reproducible results can impair the use of these measures in future (clinical) studies and even harm the credibility of the functional connectivity research field.

Examples of inconsistencies in functional connectivity literature, among others, can be found in the results reported for coherence. It is reported to be decreased in the alpha (8–13 Hz) band in AD subjects by multiple studies but some other studies have additionally reported an increase in delta (0.5–4 Hz) band coherence [18, 19] and an increase in theta (4–8 Hz) band coherence [20]. In another comparison of AD and cognitively normal patients, both the phase lag index (PLI) and imaginary coherence showed a decrease in functional connectivity in the beta (13–30 Hz) frequency band [21], where other studies showed differences in the alpha band PLI [22] and alpha [23] but also delta and theta band [24] imaginary coherence.

Some of the conflicting reports of change in functional connectivity may be explained by issues in study design such as differences in populations, sample size and (pre-)processing. In addition, the choice of functional connectivity measure could also have had an influence on the results [25]. This could be due to differences in the use of EEG properties (i.e. amplitude, phase, frequency), the chosen bandwidth and regional changes. As shown by research in other neurodegenerative diseases, different functional connectivity measures can show different regional increases or decreases in the same or different bandwidths [26, 27]. Neurodegeneration also proves to be a nonlinear process which can cause different states in different stages of the disease [28, 29]. As a result, it can be a valid procedure to look at different functional connectivity measures in different frequency bands simultaneously. However, inconsistencies can also occur due to a low reliability of the measure of functional connectivity. Large differences in test-retest reliability between measures have been reported [30, 31]. Moreover, even though a measure can be reliable, it also needs to be a valid measure of functional connectivity. An important factor influencing the validity of functional connectivity measures is the susceptibility to volume conduction [32]. Volume conduction can inflate or deflate the presented results [33], and therefore, functional connectivity measures without correction for this phenomenon are not valid measures of connectivity. This implies that the choice of functional connectivity measure can have an influence on the reproducibility, generalizability and validity of the results.

An influencing factor, briefly mentioned above, is that the diagnostic process has evolved from a mostly clinical evaluation to a biomarker-based process. Due to the presence of many comorbidities and clinical mimics of AD, results from studies that involved patients without biomarker support might have been negatively influenced. Therefore, the AD research field has adopted a new research framework [13] implementing amyloid, tau and neurodegeneration (A/T/N) criteria to ensure the results of current research are residues of the same biological process. In summary, it is important to evaluate the observed effects of AD on functional connectivity in light of the latest research framework, previously found discrepancies between test results and test-retest reliability issues of some functional connectivity measures.

This problem leads to the following question and aim of this study: which functional connectivity measures observe reproducible and valid EEG changes in AD and in which frequency bands do these changes occur? We aimed to answer these questions by designing a study in which we compared commonly used functional connectivity measures with respect to reproducibility, undesirable influence of covariates and correlation with disease severity. Data was gathered from a memory clinic to create a large population size with available EEG recordings to counter the statistical power limitations of earlier studies. The A/T/N criteria [13] were used to ensure AD-specific changes were observed. And in addition, a large second cohort was created to assess reproducibility. Other possible factors of influence, such as medication or other neurological and psychiatric disorders, were investigated. To our knowledge, no previous studies with EEG functional connectivity have been performed having such a large population size and availability of A/T/N classification.

To further evaluate the capacity of the functional connectivity measures to distinguish between different levels of disease severity, correlations with the Mini-Mental State Exam (MMSE) were estimated. Furthermore, collinearity between relative power and functional connectivity, to assess the potential influence, and the collinearity between the different functional connectivity measures were estimated. Although measures might be mathematically different, we expected that the observed variance could still be very similar between some of the measures due to shared ground principles.

## Methods

### Clinical sample

A test and a validation cohort were formed using clinical data from the clinical Amsterdam dementia cohort (ADC) in the Alzheimer Center VUmc, Amsterdam UMC, in the Netherlands [34]. All subjects visited the clinic as part of their diagnostic process during the period of 2002 to 2015 and were asked for informed consent to store their clinical data. EEGs were made as part of this process next to clinical assessment, neuropsychological test batteries, magnetic resonance imaging (MRI) or computed tomography scan (CT scan) and, when possible, assessment of amyloid burden by cerebrospinal fluid (CSF) or positron emission tomography (PET). Based on this gathered information, the final diagnosis was made by a multidisciplinary team of a neurologist, psychiatrist, neuropsychologist and radiologist during a consensus meeting according to the most recent clinical criteria. Data from the EEG recordings was used for various research purposes over time, and subjects were included based on the availability of epochs from these studies. Subjects with the clinical diagnosis of probable AD dementia [12] and subjective cognitive decline (SCD) with available EEG recordings were included.

### Cohort characteristics

For this study, when biomarkers were available, subjects were classified using the A/T/N framework [13] by using CSF amyloid beta (Aβ) 1–42, p-tau and medial temporal atrophy (MTA [35], scored from 0 to 4 which resembles no to most severe atrophy) averaged over left and right. Drift-corrected Aβ 1–42 values were used with a cut-off of 813 pg/mL [36]. This correction was applied due to the gradual increase of Innotest Aβ 1–42 CSF values over two decades of testing in the ADC, which could cause misclassification of amyloid status. When both amyloid-PET and CSF were available, the amyloid-PET was decisive. Cut-off for p-tau was set at 52 pg/mL, and the neurodegeneration cut-off was set at MTA ≥ 1 based on visual assessment of T1-weighted acquisition MRI images [37]. Furthermore, patients were categorized by Fazekas score (0–1 and ≥ 2) based on visual assessment of fluid-attenuated inversion recovery (FLAIR) images on MRI [38].

The medical history of every patient was analysed and categorized with special interest for 3 possible confounding characteristics: vascular disease, central nervous system (CNS) disease and psychiatric disease. Vascular disease included only symptomatic vascular disease such as myocardial infarction and stroke. CNS disease included any major neurologic disease but also a medical history with significant brain injury. Psychiatric disease consisted of any major active or chronic psychiatric disease. Additionally, data of the use of any medication with potential effects on the EEG (acetylcholinesterase inhibitors, benzodiazepines, anti-epileptic drugs and antidepressants) was collected.

To create a cross-sectional design with internal validation, clinically diagnosed AD and SCD subjects were randomly allocated to two cohorts. Two subset populations of cohort 1 were additionally created based on the A/T/N framework and potential interfering covariates. Subpopulation 1: SCD subjects without amyloid (CSF or PET) and tau (p-tau) pathology versus AD subjects with amyloid and tau pathology. Subpopulation 2: SCD subjects without amyloid pathology, tau pathology, neurodegeneration (MTA < 1), small vessel disease (SVD) (Fazekas < 2) or use of interfering medication versus AD subjects with amyloid pathology, tau pathology, neurodegeneration (MTA ≥ 1) and without SVD (Fazekas < 2) or use of interfering medication.

### EEG recordings

Twenty minutes of eyes-closed task-free EEG recordings was made in a standardized protocol using the 21 electrode positions of the 10–20 system. Patients sat with eyes closed in a slightly reclined chair in a sound-attenuated room. EEG technicians were alert on keeping the participants awake and to minimize artefacts. Acoustic stimuli were used when slow horizontal eye movements or slowing of the posterior alpha rhythm appeared. The sample frequency was set to 500 Hz; electrode impedance was kept below 5 kΩ with low pass filter ≤ 70 Hz, high pass filter ≥ 0.5 Hz, and no notch filter; and the average reference was used. Trained researchers visually inspected the recordings looking for artefacts and the state of alertness. Four to five artefact-free epochs of 8.192 s were visually selected for each patient. The process of the visual selection process was previously described by Gouw and colleagues [39]. In short, the visual inspection of epochs was performed by a trained EEG researcher, based on the presence of a minimum of artefacts (e.g. excessive muscle activity, eye blinks) and drowsiness. If no sufficient quality was reached, the epochs were replaced by other epochs or the EEG was excluded from analyses when insufficient epochs could be included for analyses.

### EEG analyses

The freely available ‘Brainwave’ software [40] was used to estimate functional connectivity. Mean global coherence (Coh), imaginary coherence (iCoh), phase locking value (PLV), amplitude envelope correlation (AEC), AEC with leakage correction (AEC-c), phase lag index (PLI) and weighted PLI (wPLI) were estimated in five frequency bands: broadband (0.5–30 Hz), delta (0.5–4 Hz), theta (4–8 Hz), alpha (8–13 Hz) and beta (13–30 Hz). Functional connectivity per electrode was estimated by averaging the values for each possible electrode pair per electrode (for example, the value of electrode Fp1 is the average of each potential electrode pair with electrode Fp1). The results of all electrodes were averaged to create global values. Fast Fourier transformation was used to estimate mean global relative delta, theta, alpha and beta power. The results of the different epochs were averaged for each subject.

### Functional connectivity measures

Functional connectivity measures were chosen based on their usage in AD EEG literature [2, 5, 41, 42] and test-retest reliability information [31]. The mathematical procedures to estimate these measures are described in the following paragraph in which we distinguish between measures with or without correction for volume conduction. Measures without correction are prone to signal leakage from channel to channel with the potential to deflate or inflate the connectivity estimate [33].

#### Measures without correction for volume conduction

Functional connectivity assesses functional communication between brain areas by estimating the level of synchronization of the EEG signals. To analyse statistical interdependencies, the wave-like EEG signal can be decomposed into different properties such as the frequency, amplitude or phase of the signal. The analytical signal *z*(*t*) can be described as shown in Eq. 1 where *x*(*t*) describes the real component of the time series, $\overset{\sim }{x}$(*t*) the corresponding Hilbert transform, *A*(*t*) the instantaneous amplitude and *ϕ*(*t*) the instantaneous phase.

1 $$ z(t)=x(t)+i\overset{\sim }{x}(t)=A(t){e}^{i\varphi (t)} $$

The instantaneous amplitude (or amplitude envelope) and phase can be obtained from *z*(*t*) by using, respectively, Eqs. 2 and 3.

2 $$ A(t)=\sqrt{{\left[x(t)\right]}^2+{\left[\overset{\sim }{x}(t)\right]}^2} $$

3 $$ \varphi (t)=\arctan \frac{\overset{\sim }{x}(t)}{x(t)} $$

How to obtain the Hilbert transform $\overset{\sim }{x}$ (*t*) of *x*(*t*) is shown in Eq. 4, where PV refers to the Cauchy principal value.

4 $$ \overset{\sim }{x}(t)=\frac{1}{\pi}\mathrm{PV}{\int}_{-x}^{\infty}\frac{x\left(\tau \right)}{t-\tau } d\tau $$

Coherence (Coh) [32, 43] is a functional connectivity measure which analyses synchronization in the frequency domain. Coherence is the absolute value of Coherency *c* which can be calculated (after applying the Hilbert transformation) according to Eq. 1 in Table 1. In this equation, *A* represents the instantaneous amplitude of signal 1 or 2 and *Δϕ* the instantaneous phase difference between the two signals.

**Table 1:** The equations of the used functional connectivity measures. For each functional connectivity measure, it is indicated whether they observe associations between signals based on amplitude (A), phase (P) or both (A/P)

Measure		Equation
1. Coherency	A/P	$c=\frac{\left\langle {A}_1{A}_2{e}^{i\Delta \varphi}\right\rangle }{\sqrt{\left\langle {A}_1^2\right\rangle \left\langle {A}_2^2\right\rangle }}$
2. Phase locking value/phase coherence	P	$R=\left|\left\langle {e}^{i\Delta {\varphi}_{ij}(t)}\right\rangle \right|=\left|\frac{1}{N}\sum \limits_{k=0}^{N-1}{e}^{i\Delta \varphi \left({t}_k\right)}\right|$
3. Amplitude envelope	A	$A(t)=\sqrt{{\left[x(t)\right]}^2+{\left[\overset{\sim }{x}(t)\right]}^2}$
4. Imaginary coherence	A/P	$\mathit{\operatorname{Im}}\left\{c\right\}=\frac{\left\langle {A}_1{A}_2s\mathrm{in}\Delta \varphi \right\rangle }{\sqrt{\left\langle {A}_1^2\right\rangle \left\langle {A}_2^2\right\rangle }}$
5. Phase lag index	P	PLI = |〈*sign*[sin(*∆φ*(*t*_*k*_))]〉|
6. Weighted phase lag index	P	$\mathrm{wPLI}\equiv \frac{\mid E\left\{\mathfrak{I}(Z)\right\}\mid }{\mathrm{E}\left\{\mathfrak{I}\left(\mathrm{Z}\right)\right\}}=\frac{\mid E\left\{\left|\mathfrak{I}(Z)\right|\mathit{\operatorname{sign}}\left(\mathfrak{I}(Z)\right)\right\}\mid }{E\left\{\left|\mathfrak{I}(Z)\right|\right\}}$

The stability of the phase difference between two time series can be estimated by using Eq. 2 in Table 1 and in literature by it has been termed as the phase locking value (PLV) [44] or the phase coherence [45]. Where the phase coherence uses the Hilbert transform prior to Eq. 2 in Table 1, the PLV uses a wavelet-based analysis. Literature has, however, shown that both approaches produce similar results [46].

The amplitude envelope correlation (AEC) [47] is an amplitude-based measure which estimates the Pearson correlation between the envelopes of the amplitudes of time series (Eq. 3 in Table 1). The amplitude envelopes are calculated by using the Hilbert transform of the time series.

#### Measures with correction for volume conduction

Several methods have been developed to correct for volume conduction. The corrected amplitude envelope correlation (AEC-c) [48] uses pair-wise orthogonalization prior to the AEC calculations described in the ‘Measures without correction for volume conduction’ section. The averaged result of a pair-wise orthogonalization in both directions, *X* to *Y* and *Y* to *X*, was used.

The imaginary coherence (iCoh) [49] can be estimated with the imaginary part of coherency according to Eq. 4 in Table 1. Where the imaginary coherence is also based on the amplitude of the signal, the phase lag index (PLI) [21] is a solely phase-based measure with correction for volume conduction. It estimates the asymmetry of the distribution of phase differences *Δφ*(*t*) between time series. Yielding low values for median phase differences of 0 mod π. It can be estimated according to Eq. 5 in Table 1. Due to the discontinuity of the index, the PLI may be hindered by small perturbations, around a phase difference of 0 mod π, which cause phase lags to turn into leads and vice versa. The weighted phase lag index (wPLI) [50] corrects for this phenomenon by using the magnitude of the imaginary component of the cross-spectrum as a weight for the phase lags (Eq. 6 in Table 1) where $\mathfrak{I}(Z)$ is the imaginary component (sin*∆φ*) of signal *Z*. Unfortunately, this measure depends upon both the consistency and the magnitude of the phase difference.

### Statistical analysis

All statistical analyses were performed using SPSS statistics software (version 24.0.0.1). Available demographic and medical characteristics were described, and differences between SCD and AD subjects in both cohorts were assessed by independent *t* test, chi-square test, Fisher’s exact test or Mann-Whitney *U* test where appropriate. Normality of distribution of the variables was checked by histograms and Q-Q plots.

Distribution of the functional connectivity measures was also checked by histograms and Q-Q plots, and when appropriate, variables were log transformed. Differences in functional connectivity measures between AD and SCD subjects were determined by two models of analysis of (co)variance (AN(C)OVA). Model 1 applied correction for the covariates age and sex. Model 2 corrected for age, sex and the relative power of the bandwidth in which the functional connectivity was measured. ANOVA on ranks [51] was performed for variables that could not be successfully log transformed. After each of the ANOVA models, the effect size was estimated by Cohen’s *d* [52]. In addition, various demographic and medical characteristics were separately added as a covariate to model 1 to check for any interfering effects. To observe regional reproducibility, we have averaged the individual channels into 4 regions: frontal (channels Fp1, Fp2, F3, F4, F7, F8, Fz), temporal (channel T3, T4, T5, T6), central (channel C3, C4, Cz) and parieto-occipital (P3, P4, Pz, O1, O2). ANCOVA model 1 (with correction for age and sex) was repeated for these regional values over each bandwidth in cohort 1 (including the 2 subpopulations) and cohort 2.

False discovery rate (FDR) [53] correction was applied to *p*-values of the demographic and medical characteristic comparisons between the SCD and AD groups. FDR correction was furthermore applied to the *p-*values of the ANOVA models correcting for the multiple testing in different bandwidths of each functional connectivity measure. Due to the expected presence of high collinearity between the tested functional connectivity measures (see the ‘Collinearity between functional connectivity measures’ section), the number of hypotheses tested in the FDR correction was set to 5 (the number of tested bandwidths). This choice was also made due to the use of a validation cohort which will in turn also reduce the number of false positive results. *p*-values shown were FDR corrected, and a threshold of *p* < 0.05 was maintained.

Correlations between global values of the different functional connectivity markers were explored, and the level of collinearity was assessed. In addition, correlations between functional connectivity and relative power were investigated. The correlations were investigated with Pearson and Spearman correlations where appropriate.

## Results

### Demographic and medical cohort characteristics

Two cohorts were created to assess cross-sectional differences in functional connectivity. Cohort 1 consisted of 197 SCD and 214 AD subjects, whereas cohort 2 consisted of 202 SCD and 196 AD subjects. Available characteristics of both cohorts are shown in Table 2. Most notable was the significant difference in age between SCD and AD subjects in both cohorts. SCD subjects were younger in both cohorts (mean difference (Δmean) of 5 and 9 years in cohorts 1 and 2, respectively). No significant differences were found in medical history between SCD and AD subjects. Significant differences in MMSE, CSF, PET and MRI results were in concordance with the SCD and AD diagnoses. Across cohorts, SCD subjects had a slightly higher MMSE in cohort 2 (Δmedian = 1). AD subjects had a slightly higher MMSE (Δmedian = 1) and MTA (Δmedian = 0.5) and lower total tau (Δmean = 4) in cohort 2.

**Table 2:** Characteristics of subjective cognitive decline (SCD) and dementia due to Alzheimer’s disease (AD) subjects in both cohorts. The count (*n*), mean or median with percentage (%), standard deviation (SD) or interquartile range (IQR) are shown for each variable. *MMSE* Mini-Mental State Exam, *CNS* central nervous system, *MTA* Medial Temporal Atrophy score, *Aβ 1–42* amyloid beta 1–42, *t-tau* total tau, *p-tau* phosphorylated tau

	Cohort 1		Cohort 2	
Characteristic	SCD *n* = 197	AD *n* = 214	SCD *n* = 202	AD *n* = 196
Female (*n*, %)	76 (39%)	104 (49%)	89 (44%)	94 (48%)
Age (mean, SD)	62 ± 8	67 ± 8^a^	60 ± 10	69 ± 10^a^
MMSE (median, IQR)	28 (27–29)	21 (17–24)^a^	29 (28–30)^b^	22 (18–25)^a,b^
Vascular disease (*n*, %)	34 (17%)	40 (19%)	35 (17%)	32 (16%)
CNS disease (*n*, %)	20 (10%)	14 (7%)	22 (11%)	19 (10%)
Psychiatric disease (*n*, %)	6 (3%)	8 (4%)	5 (2%)	1 (1%)
Antidepressants (*n*, %)	20 (10%)	26 (12%)	16 (8%)	19 (10%)
Benzodiazepines (*n*, %)	11 (6%)	12 (6%)	18 (9%)	15 (8%)
Anti-epileptic drugs (*n*, %)	4 (2%)	3 (1%)	4 (2%)	3 (2%)
Acetylcholinesterase inhibitors (*n*, %)	0 (0%)	16 (7%)^a^	1 (0%)	18 (9%)^a^
**MRI**	*n* = 176	*n* = 199	*n* = 175	*n* = 156
MTA (median, IQR)	0 (0–0.5)	1 (0.5–2)^a^	0 (0–0.5)	1.5 (1–2.5)^a,b^
Fazekas (median, IQR)	1 (0–1)	1 (0–1)	0 (0–1)	1 (0.5–1)^a^
**Cerebrospinal fluid**	*n* = 183	*n* = 169	*n* = 120	*n* = 141
Aβ 1–42 (mean, SD)	1031 ± 268	663 ± 136^a^	1090 ± 197	694 ± 162^a^
t-tau (mean, SD)	302 ± 214	731 ± 413^a^	267 ± 119	610 ± 321^a,b^
p-tau (mean, SD)	50 ± 23	89 ± 35^a^	47 ± 16	85 ± 38^a^
**Amyloid-PET**	*n* = 32	*n* = 34	*n* = 11	*n* = 17
Positive PET (*n*, %)	12 (38%)	34 (100%)^a^	3 (27%)	14 (82%)^a^

^a^Significant differences between SCD and AD subjects within cohorts (*p* < 0.05)

^b^Significant differences in the same diagnostic group across cohorts (e.g. SCD versus SCD and AD versus AD) (*p* < 0.05)

Subpopulations were formed based on the availability of CSF and amyloid-PET biomarkers. In subpopulation 1, all AD subjects with positive amyloid and tau biomarkers (*n* = 135; A+T+) and SCD subjects without evidence for AD, thus with negative amyloid and tau biomarkers (*n* = 97; A−T−), were selected. Even more strict criteria were used additionally for subpopulation 2, excluding patients with potential interfering co-medication or SVD and selection based on MTA score as a marker of neurodegeneration (A+T+N+ for AD; A−T−N− for SCD). This resulted into 71 SCD and 41 AD subjects from cohort 1.

### Functional connectivity

**Fig. 1:**
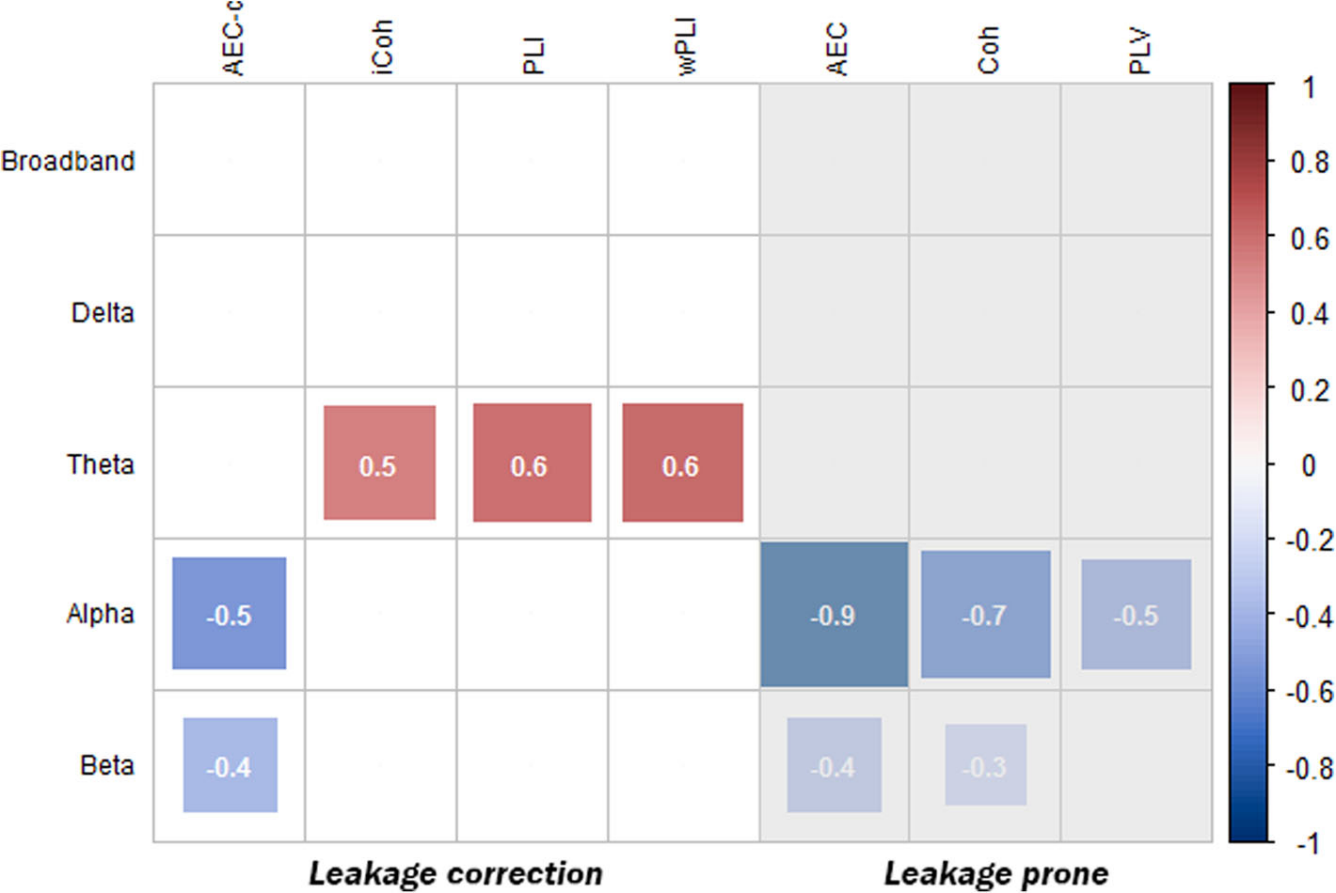
Summary of observed differences in ANOVA model 1, shown as effect size (Cohen’s *d*), between AD and SCD subjects for each of the functional connectivity measures and bandwidths. The significant effect sizes of the comparisons made in the entire cohort 1 (*n* = 411) are shown. Effects that could not be reproduced in the subset populations or cohort 2 were left out. Red blocks represent a higher and blue blocks a lower level of functional connectivity in AD subjects compared to SCD subjects. The size of the blocks and the number shown in the blocks represent the size of the effect. Results of functional measures susceptible to signal leakage are shaded in grey

Differences in global functional connectivity between SCD and AD subjects in cohort 1 were estimated by ANOVA model 1. A summary of the observed effect sizes is shown in Fig. 1. A more detailed description, including mean value, standard deviation and *p-*value, of the significant effects shown in Fig. 1 can be found in supplementary Table 1. Figure 1 only shows the global effects that were both significant in cohort 1, subpopulation 2 and validation cohort 2.

Clear differences between functional connectivity measures can be observed in Fig. 1. Firstly, the AEC-c, AEC, coherence and PLV showed lower functional connectivity in the alpha (*d* = − 0.5; *d* = − 0.9; *d* = − 0.7; *d* = − 0.5, respectively) and beta (*d* = − 0.4; *d* = − 0.4; *d* = − 0.3; no effect of the PLV, respectively) frequency bands in AD subjects. Secondly, the imaginary coherence, PLI and wPLI observed higher functional connectivity in the theta frequency band in AD subjects (*d* = 0.5; *d* = 0.6; *d* = 0.6, respectively). Thirdly, the delta frequency band and broadband functional connectivity did not show robust group differences. The largest difference between the AD and SCD subjects was observed in the uncorrected AEC in the alpha band (Cohen’s *d* = − 0.90, *p* = 8.5 × 10^−18^, model 1). This effect remained the largest when the population was selected based on amyloid and phosphorylated tau (subpopulation 1, *d* = − 0.83, *p* = 1.8 × 10^−9^) or the complete A/T/N criteria, exclusion of SVD and potential interfering medication (subpopulation 2, *d* = − 1.03, *p* = 4.6 × 10^−7^). In cohort 2, this difference was validated to be the largest of the tested measures (*d* = − 0.68, *p* = 6.2 × 10^−11^).

**Fig. 2:**
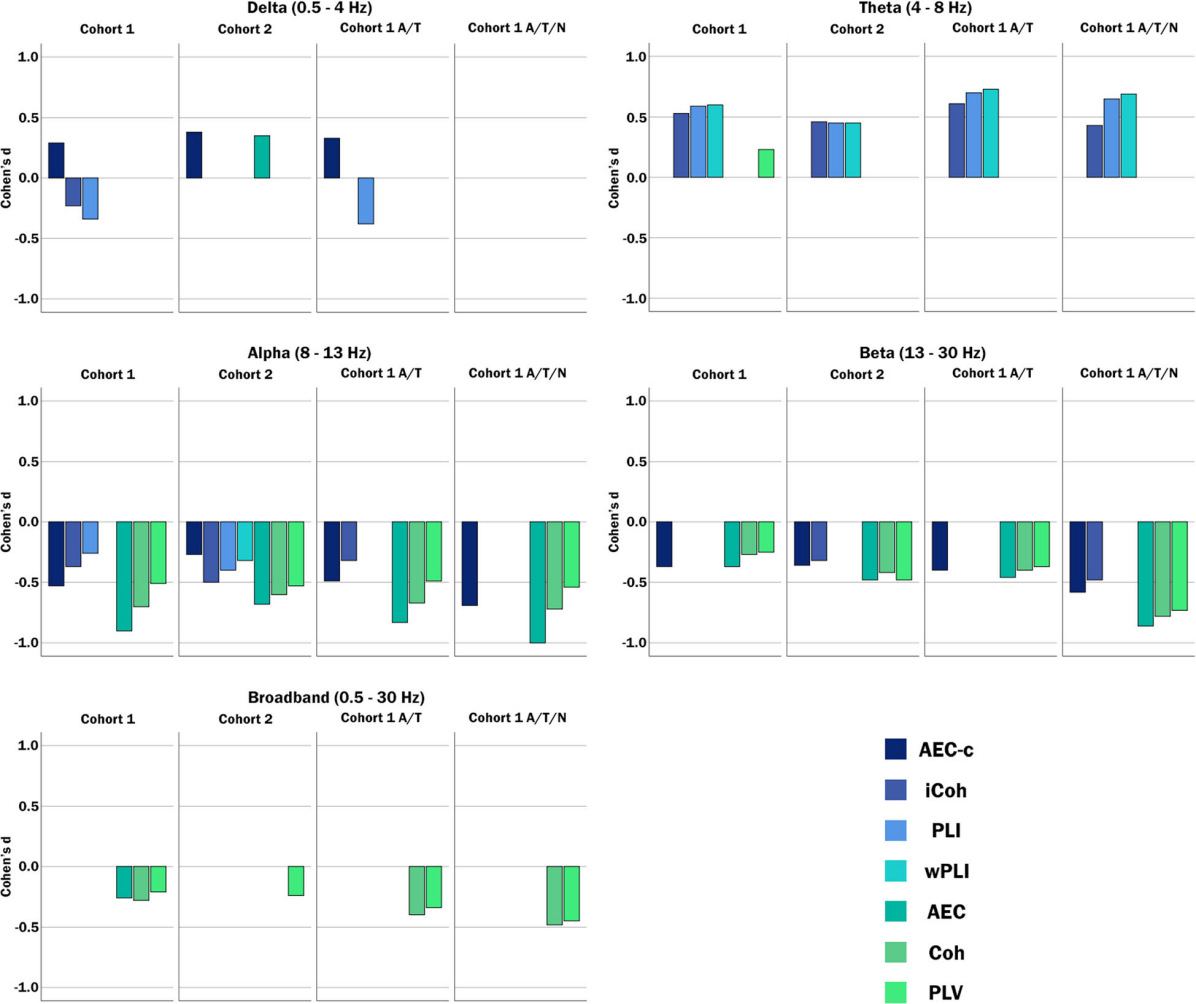
The difference in functional connectivity between SCD and AD subjects per bandwidth is shown for each cohort and subpopulation. Significant effect sizes are shown in Cohen’s *d* estimated by GLM model 1. Cohort 1: all SCD and AD subjects in cohort 1. Cohort 2: all SCD and AD subjects in cohort 2. Cohort 1 A/T: amyloid-negative/tau-negative SCD versus amyloid-positive/tau-positive AD subjects from cohort 1 (subpopulation 1). Cohort 1 A/T/N: amyloid-negative/tau-negative/MTA < 1 SCD versus amyloid-positive/tau-positive/MTA ≥ 1 AD subjects from cohort 1, excluding any patients with Fazekas > 1 and any potential interfering medication (subpopulation 2)

The significant effects observed in cohort 1, cohort 2 and both subpopulations are shown in Fig. 2. Although minor differences in observed effects and effect sizes are present, most effects observed in the entire cohort 1 are generally similar to the effects in the subpopulations and the validation cohort. Notable differences across cohorts were a reproducible effect on the delta band AEC-c in cohorts 1 and 2 and subpopulation 1 (A/T) but not 2 (A/T/N) (see topographic distributions below) and a reproducible effect of the alpha band PLI and wPLI in cohorts 1 and 2 but not in subpopulations 1 and 2.

In order to look at the topographic distribution of the observed effects, the AEC-c and PLI were specifically selected because these measures correct for volume conduction and showed reproducible global effects. The topographic distributions of AEC-c and PLI in cohort 1 are shown in supplementary Figure 1. The reproducibility of the regional effects on the AEC-c and PLI is shown in supplementary Figure 2A and 2B. For all regions, a reproducible increase in theta band PLI and a decrease in alpha band AEC-c were found. The effect of the PLI was the strongest in the temporal channels (*d* = 0.66, *p* = 9.2 × 10^−11^), and the effect of the AEC-c was the strongest in the temporal (*d* = − 0.56, *p* = 3.2 × 10^−8^) and parieto-occipital (*d* = − 0.56, *p* = 2.9 × 10^−8^) channels. Furthermore, the AEC-c showed reproducible effects in the delta band AEC-c of the central channels and beta band AEC-c of the frontal, temporal and parieto-occipital channels.

### Influence of medication and medical history

The influence of potential covariates (medication: acetylcholinesterase inhibitors, benzodiazepines, anti-epileptic drugs, antidepressants; medical history: CNS disease, psychiatric disease, presence of SVD) was investigated by adding the variables to model 1. The only significantly observed (small) effects were a decrease in beta band PLI, wPLI and PLV (*d* = − 0.22, *p* = 0.027; *d* = − 0.22, *p* = 0.028; *d* = − 0.24, *p* = 0.020) and an increase in delta band wPLI (*d* = − 0.22, *p* = 0.031) associated with antidepressants. Furthermore, a decrease in beta band PLV (*d* = − 0.24, p = 0.020) was associated with the presence of SVD. Adding these covariates to model 1 did not change the observed effects between AD and SCD subjects.

### Effect of relative power

**Fig. 3:**
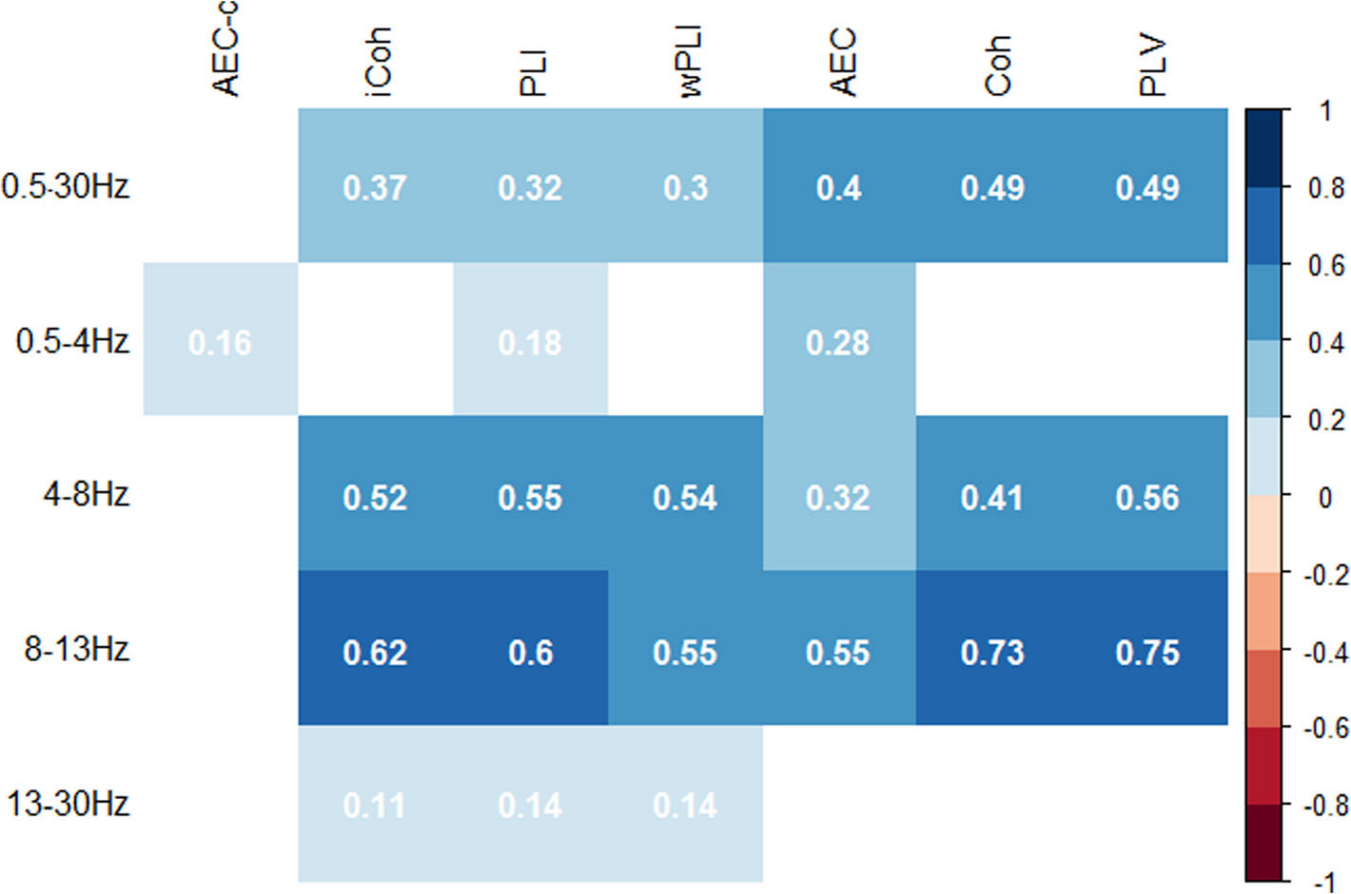
Correlation of different functional connectivity measures with the relative power in the corresponding frequency band. The functional connectivity measures are shown on the *x*-axis and the band power on the *y*-axis. Only significant correlations are shown, indicated by the correlation coefficient (*r*) which is also indicated by the colour gradient from *r* = − 1 (dark red) to *r* = + 1 (dark blue)

To evaluate the relation of relative power with different functional connectivity measures, Pearson and Spearman correlations were estimated between these variables. The correlations were calculated between the functional connectivity in each frequency band and the relative power in that frequency band (e.g. correlation between alpha band AEC-c and relative alpha power). The results can be found in Fig. 3.

Various functional connectivity measures showed very high correlations with the relative power in the corresponding bandwidth. These effects were most pronounced in the theta and alpha bands. The two highest correlations were between the alpha band coherence and PLV with the relative alpha power (*r* = 0.73, *p* = 4.4 × 10^−67^; *r* = 0.75, *p* = 4.0 × 10^−72^, respectively). In contrast to the other functional connectivity measures and apart from a small effect (*r* = 0.16, *p* = 0.001) in the delta frequency band, the AEC-c was not correlated to the relative power.

**Fig. 4:**
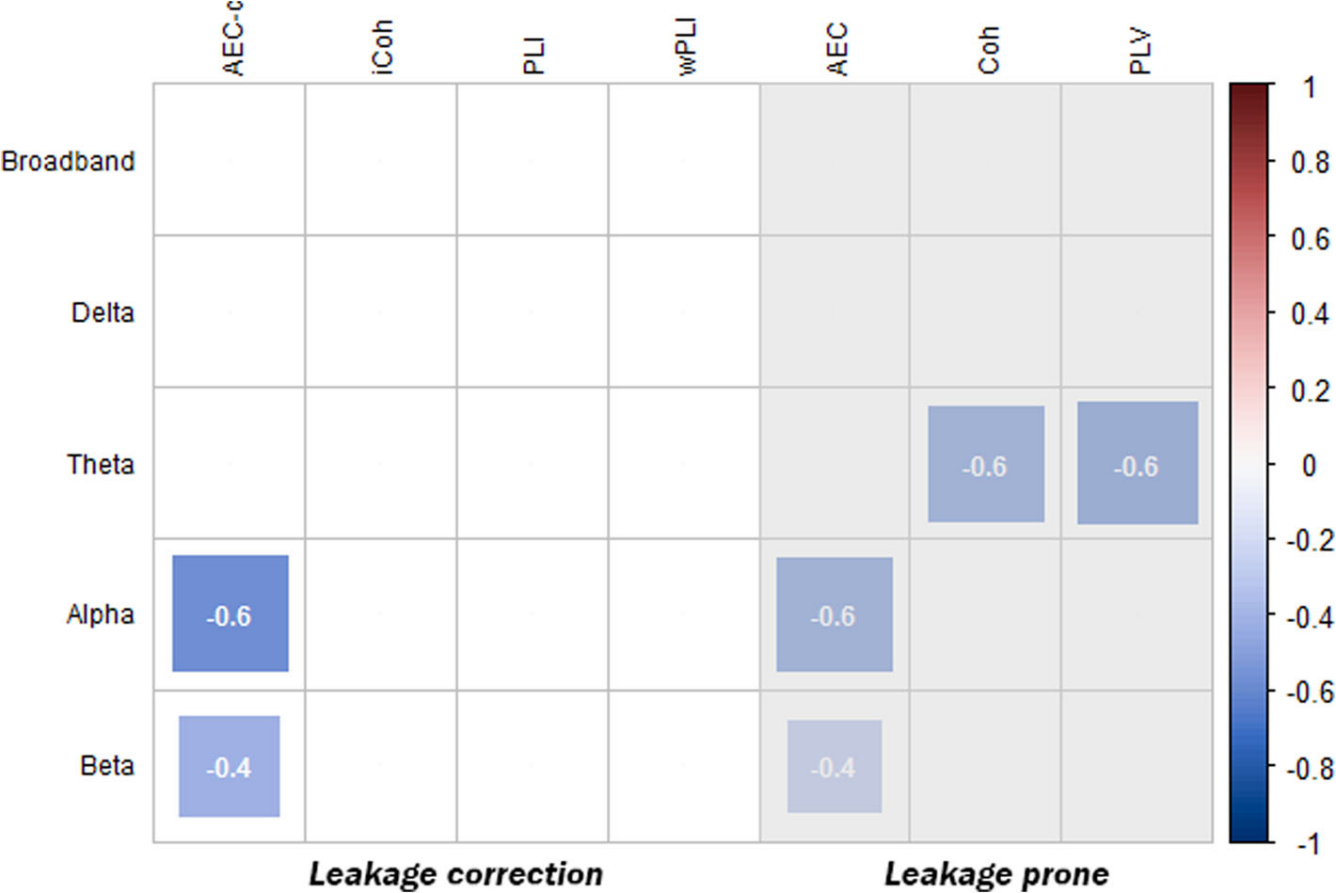
Summary of observed differences in ANOVA model 2, shown as effect size (Cohen’s *d*), between AD and SCD subjects for each of the functional connectivity measures and bandwidth. The significant effect sizes of the comparisons made in the entire cohort 1 (*n* = 411) are shown. Effects that could not be reproduced in the subset populations or cohort 2 were left out. Red blocks represent a higher and blue blocks a lower level of functional connectivity in AD subjects compared to SCD subjects. The size of the blocks and the number shown in the blocks represent the size of the effect. Results of functional measures susceptible to signal leakage are shaded in grey

To observe how much of the variance of the effects between AD and SCD subjects observed in ANOVA model 1 could be explained by changes in relative power, the analyses were repeated with additional correction for relative band power (model 2). In line with the analysis of model 1, shown in Fig. 1, the results of model 2 are shown in Fig. 4. Again, only the effects of cohort 1, which were significant in cohort 1, subpopulation 2 and validation cohort 2, are shown. More detailed results of Fig. 4 can be found in supplementary Table 2.

In comparison with ANOVA model 1, the effects observed by the AEC-c remained stable in model 2 (model 1: alpha band *d* = − 0.53, *p* = 1.24 × 10^−7^; beta band *d* = − 0.37, *p* = 2.31 × 10^−4^; model 2: alpha band *d* = − 0.57, *p* = 1.9 × 10^−8^; beta band *d* = − 0.42, *p* = 3.3 × 10^−5^). The AEC effect also remained stable in the beta band but decreased in the alpha band compared to model 1. The observed effects of the coherence (alpha and beta bands) and PLV (alpha band) in model 1 disappeared in model 2. The theta band effects of the imaginary coherence, PLI and weighted PLI disappeared as well. In contrast, where no theta band effect was found with the coherence and PLV in model 1, these measures showed a reduced theta band functional connectivity in AD subjects, with the largest effect for the PLV (*d* = − 0.62, *p* = 1.0 × 10^−9^), in model 2.

### Disease severity and functional connectivity

In Fig. 1, the functional connectivity measures were shown that show consistent differences between SCD and AD. However, a functional connectivity measure which is indicative for disease severity would be even more valid and clinically relevant. Therefore, Pearson correlation coefficients were calculated between the valid connectivity measures in Fig. 1 and disease severity represented by the MMSE score (Table 3). Of the 12 observed functional connectivity measures from model 1, only 4 showed correlations with disease severity in the AD subjects, with the largest, but modest, effects of the AEC-c (*r* = 0.14, *p* = 0.0054) in the alpha band and the AEC (*r* = 0.13, *p* = 0.0073) in the beta band.

**Table 3:** Pearson correlation coefficients (*r*) between functional connectivity (FC) and disease severity represented by MMSE. Correlations were estimated for the entire population (cohorts 1 and 2) and the AD subjects only. Bold *r* values indicate a significant (*p* < 0.05) correlation

FC measure versus MMSE	All subjects (***n*** = 809)		AD only (***n*** = 410)	
	***r***	*p-*value	***r***	*p-*value
**Theta (4–8 Hz)**				
iCoh	**− 0.13**	1.41 × 10^−4^	− 0.06	0.23
PLI	**− 0.13**	2.79 × 10^−4^	− 0.06	0.23
wPLI	**− 0.13**	1.71 × 10^−4^	− 0.06	0.21
**Alpha (8–13 Hz)**				
AEC-c	**0.21**	3.2 × 10^−9^	**0.14**	0.0054
AEC	**0.35**	6.1 × 10^−25^	**0.14**	0.0065
Coh	**0.27**	6.4 × 10^−15^	0.04	0.40
PLV	**0.23**	7.3 × 10^−11^	0.08	0.13
**Beta (13–30 Hz)**				
AEC-c	**0.21**	1.8 × 10^−9^	0.08	0.12
AEC	**0.26**	1.0 × 10^−13^	**0.13**	0.0073
Coh	**0.22**	3.1 × 10^−10^	**0.12**	0.015

### Collinearity between functional connectivity measures

Some of the effects observed by functional connectivity markers in ANOVA model 1 were very similar in size. To observe whether they measured similar differences between subjects, Pearson correlations between all functional connectivity measures were estimated. In addition, due to the high correlations with relative power observed in the ‘Effect of relative power’ section, the analyses were repeated with statistical correction for relative power. Results of the theta, alpha and beta bandwidths can be found in supplementary Figure 3A-C. These figures show similar clusters of correlating functional connectivity markers in the different bandwidths. High correlations between the imaginary coherence, PLI, wPLI and PLV are evident. In contrast, the AEC-c showed only strong correlations with its uncorrected version.

## Discussion

### Main outcomes

By using large well-characterized cohorts and the most recent NIA-AA research framework for Alzheimer’s disease [13], we aimed to find the most reproducible and robust changes of EEG functional connectivity in Alzheimer’s disease. The most reproducible and robust observed effects were decreases in the alpha and beta band AEC-c functional connectivity in the AD subjects. Furthermore, sensitivity of different functional connectivity measures appeared to be highly bandwidth specific, as most volume conduction-corrected phase-based measures showed reproducible increased functional connectivity in the theta band, whereas amplitude-based or more general connectivity measures showed decreased functional connectivity in the alpha and beta bands.

The conclusion that the decreases in alpha and beta band AEC-c were the most reproducible and robust functional connectivity estimates in AD is based upon the following observations. First, the AEC-c retained reproducible results in the test and validation cohorts and these effects were amplified when the analyses were repeated in a subpopulation of patients fulfilling the A/T/N criteria. This implies that these effects are (Alzheimer’s) disease specific. Secondly, the observed effects were robust. The effects of the alpha and beta band AEC-c were not influenced by relative band power, several demographic variables, (co)morbidities or interfering medication. Thirdly, the AEC-c was not only a disease-specific marker of AD but also correlated with disease severity. In AD subjects, lower alpha band AEC-c values modestly correlated with lower MMSE scores. Lastly, the AEC-c has been shown to be a reliable functional connectivity measure in previous test-retest reliability research [31]. Colclough and colleagues have tested all the functional connectivity measures used in our study on group-level repeatability and within-subject and between-subject consistency. Overall, the AEC-c showed the most consistent results compared to other measures corrected for volume conduction.

We propose the effects in the frequency bands and functional connectivity measures as shown in Fig. 1 as most reproducible. These effects remained present in large cohorts (i.e. high statistical power) but also after selection for AD-specific biomarkers in a smaller subpopulation. It is however most preferable to use functional connectivity measures that are insensitive to volume conduction or change in relative power. When looking at the summarized effects of all functional connectivity measures between SCD and AD, two major trends between different measures can be observed. Volume conduction-corrected phase-based measures (PLI, wPLI and iCoh) showed an increase in theta band functional connectivity, whereas amplitude or more mixed connectivity-based measures (AEC(-c), coherence, PLV) showed decreases in the alpha and beta bands. Therefore, future studies could consider using both a phase-based measure in the theta band and an amplitude-based measure in the alpha or beta band for their study or trial design. More research is needed to investigate the underlying processes of these distinctly different effects in phase and amplitude connectivity in AD. Phase- and amplitude-based functional connectivity measures could potentially capture different aspects of different patho- or neurophysiological processes. A review by Engel and colleagues proposed that amplitude- (or envelope) based connectivity has a close relation with the structural network and is relatively robust against state changes [54]. Phased-based connectivity appeared to be less related to the structural network and showed a stronger state dependence. On the other hand, recent literature has also shown that modulation of phase-amplitude coupling in long-range circuits may be highly relevant in cognitive functioning [55], indicating that amplitude and phase connectivity could be two interacting modalities. Changes in phase-based connectivity could affect amplitude-based connectivity (in the same or other regions) and vice versa.

### Main outcomes compared to literature

We have compared our results with previous findings in literature. The decrease in alpha and beta band AEC-c is supported by previous MEG and EEG studies [42, 56]. Our regional analyses showed a widespread decrease of alpha and beta band AEC-c, with the exception of the beta band AEC-c in the central channels. In comparison, an EEG study by Nuñez and colleagues [56] compared the alpha and beta AEC-c in healthy controls with AD subjects and found a similar widespread decrease in alpha and beta band AEC-c. MEG has a higher spatial resolution than EEG, offering a potential explanation why in a MEG study between elderly controls and AD subjects, Koelewijn and colleagues found the most outspoken decreases in beta band AEC-c specifically in the bilateral (middle-) superior temporal and parietal cortex [42] instead of a general widespread decrease. The sensitivity of the AEC-c in the alpha and beta bandwidths might not be surprising because they are in line with an earlier methodological study by Hipp and colleagues [48] which has shown the AEC-c to be the most sensitive to changes in these bandwidths.

A decrease in alpha band coherence in AD is widely known in the present literature [2, 41] and was reproduced in our study. A decrease or increase in delta and theta band coherence was reported by some articles [18–20] but could not be reproduced in either of our cohorts and subpopulations. Various other studies could also not reproduce these results [2, 41]. Potential explanations include small sample sizes, poor inclusion criteria and suboptimal control populations.

A global and widespread increase in theta band phase-based measures with correction for volume conduction (PLI, wPLI, iCoh) is in line with some previous studies [24, 57]; however, we could not reproduce a decrease in the alpha band [5, 23]. We did observe some alpha band effects with the phase-based measures in the entire cohort but these effects disappeared when the NIA-AA research criteria [13] are applied and potential interfering factors are eliminated. The effects could therefore potentially be caused by common co-pathology such as vascular disease [58] or commonly missed diagnoses such as Lewy body dementia (DLB) [59]. This could influence the results as shown by a recent study by van der Zande and colleagues [60] where profound lower alpha band PLI values for DLB subjects compared to AD subjects were found. Another explanation could be the low test-retest reliability of the phase-based functional connectivity measures [31].

### Effect of relative power

Many of the tested functional connectivity measures had a high correlation with relative power in the corresponding frequency band. Only the AEC-c seems to be independent of relative power in most of the frequency bands. This was also represented in ANOVA model 2, where the effects observed by the AEC-c were not affected by a statistical correction to relative power. The high correlation of the AEC, coherence, PLV and, to lesser extent, the imaginary coherence with relative power is possibly due to the susceptibility of these measures to volume conduction effects [32, 61] and potentially due to changes in signal to noise ratio (SNR). An increase in relative power (and subsequently signal to noise ratio) is known to be correlated with an increase in volume conduction [32, 62]. Another consideration is that previous research has shown that there is a physiological relation between oscillatory activity and functional connectivity. Tewarie and colleagues have, for example, shown a relation between the amplitude of a signal and the dynamic functional connectivity measured by phase-difference derivative (PDD) [63]. This study showed that the PDD and the amplitude positively correlate in resting state MEG, sensorimotor task MEG and data based on a neuronal model. Nonetheless, it remains unclear what the exact relations are between oscillatory activity and the different functional connectivity measures applied in our study. The correlation of functional connectivity measures without correction for volume conduction with relative power was the strongest in the alpha frequency band, which implies that interpretation of results within this functional connectivity band should be made with caution. One of the caveats of applying statistical correction for relative power in cross-sectional studies is, however, potential overcorrection due to group effects. Due to the difference in relative power and functional connectivity between SCD and AD subjects, it is likely that these measures show similar relationships. Statistical correction for the strong effect of an increased global theta power in AD, for example, may diminish the observed effect of volume conduction-corrected measures such as the PLI and wPLI in the theta band. Contrastingly, the absence of a correlation of relative power with the AEC-c does imply the presence of a robust effect.

### Collinearity between functional connectivity measures

High correlations between the different functional connectivity measures have been found. This might not be surprising because, in principle, many were designed to explain similar phenomena. In our test setting, for example, high correlations were found between the imaginary coherence, PLI and wPLI. With *r* values around 0.9 and higher, there is very high collinearity between these measures. Although the theoretical backgrounds and methods of calculation differ [21, 49, 50], these measures yielded almost identical results when comparing AD versus SCD. The AEC-c only showed fairly high correlations with its uncorrected counterpart (AEC).

### Disease severity and functional connectivity

On average, the significant correlations between the level of functional connectivity and the MMSE score were weak. There are several potential explanations for this observation. The MMSE is a general test of cognition but emphasizes on memory function whilst AD patients also suffer from deficits in, for example, language or executive functioning. Other neuropsychological tests might have observed other effects. Furthermore, correlations were made between global levels of connectivity where regional connectivity could have stronger correlations with dysfunction in certain cognitive domains [23].

### Strengths, limitations and future directions

The major strengths of our study were the large well-characterized sample size, a study design with internal validation, the use of the most recent NIA-AA AD research framework [13] with the availability of multiple biomarkers, rigorous correction for potential confounders and relative power, and the fact that multiple commonly used functional connectivity measures were tested. Previous studies do not only differ in the use of certain functional connectivity measures but also in epoch selection (no selection, visual selection or automatic selection), artefact rejection and other pre-processing steps. As there is no consensus on which method to use, we preferred to only do a protocolized visual selection by trained and experienced technicians based on our previous experience. To our knowledge, no other pre-processing methods have been proven to be superior in 21-channel EEG analyses. Potential limitations include the absence of an external validation cohort. Additionally, we have not investigated all available functional connectivity measures but made a selection based on commonly used measures and the previously reported intra-subject reliability of these measures [31]. It should also be considered that the reproducibility of functional connectivity in eyes-open EEG or task EEG data was not investigated. Moreover, additional research is needed to investigate the discriminative value of these reproducible measures between disease stages or the association with more specific tasks of different cognitive domains.

Future research, such as clinical trials or connectivity and network studies, could yield false positive or negative results by using unreliable functional connectivity measures or by using these measures in an invalid bandwidth. The results of this study provide information on which changes in functional connectivity are reproducible for Alzheimer’s disease and to what extend they correlate with disease severity. This enables future studies to narrow-down and predefine functional connectivity measures as outcome measures.

## Conclusion

In this large cross-sectional cohort study of well-characterized patients with AD and SCD, the AEC-c in the alpha and beta bands showed the most reproducible changes in functional connectivity, independent of various influencing factors, and strongest correlation with disease severity. Phase-based measures, with correction for volume conduction, observed strong effects in the theta band. These results might offer our research field some directions in solving the ‘reproducibility crisis’.

## Supplementary information

**Additional file 1: Supplementary Table 1.** Difference in functional connectivity (FC) between SCD and AD subjects estimated by ANOVA model 1 (correction for age and gender). **Supplementary Table 2.** Difference in functional connectivity (FC) between SCD and AD subjects estimated by ANOVA model 2 (correction for age, gender and global relative power). **Supplementary Figure S1.** Topographical distribution of the median difference in Z-score of the PLI and AEC-c between the SCD and AD subjects. **Supplementary Figure S2A & 2B.** Summary of observed differences in ANOVA model 1, shown as effect size (Cohen’s d), between AD and SCD subjects in different regions for the AEC-c and PLI in each bandwidth. **Supplementary Figure S3A-C.** Correlation coefficients (r) between functional connectivity measures in the theta (A), alpha (B) and beta (C) bandwidths. In each figure, the coefficients on the right are corrected for changes in relative power and the coefficients on the left are the uncorrected values.

## Data Availability

The datasets used and/or analysed during the current study are available from the corresponding author on reasonable request.

## Abbreviations

AD: Alzheimer’s disease
SCD: Subjective cognitive decline
EEG: Electroencephalography
Coh: Coherence
iCoh: Imaginary coherence
PLV: Phase locking value
AEC: Amplitude envelope correlation
AEC-c: AEC with leakage correction
PLI: Phase lag index
wPLI: Weighted PLI
CSF: Cerebrospinal fluid
Aβ: Amyloid beta
t-tau: Total tau
p-tau: Phosphorylated tau
MMSE: Mini-Mental State Exam
CNS: Central nervous system
MTA: Medial Temporal Atrophy score
PET: Positron emission tomography
MRI: Magnetic resonance imaging
