# Supplementary material for: Reproducibility of EEG functional connectivity in Alzheimer’s disease
Source: Alzheimers Res Ther. 2020 Jun 3;12:68. doi: 10.1186/s13195-020-00632-3 (PMC7271479; doi:10.1186/s13195-020-00632-3)
Supplement: Supplementary file 1 — Additional file 1: Supplementary Table 1. Difference in functional connectivity (FC) between SCD and AD subjects estimated by ANOVA model 1 (correction for age and gender). Supplementary Table 2. Difference in functional connectivity (FC) between SCD and AD subjects estimated by ANOVA model 2 (correction for age, gender and global relative power). Supplementary Figure S1. Topographical distribution of the median difference in Z-score of the PLI and AEC-c between the SCD and AD subjects. Supplementary Figure S2A & 2B. Summary of observed differences in ANOVA model 1, shown as effect size (Cohen’s d), between AD and SCD subjects in different regions for the AEC-c and PLI in each bandwidth. Supplementary Figure S3A-C. Correlation coefficients (r) between functional connectivity measures in the theta (A), alpha (B) and beta (C) bandwidths. In each figure, the coefficients on the right are corrected for changes in relative power and the coefficients on the left are the uncorrected values. [file 13195_2020_632_MOESM1_ESM.zip › Supplementary_materials_21_4_2020.docx]

Supplementary materials

**Supplementary tables & figures**

*Supplementary table 1: Difference in functional connectivity (FC) between SCD and AD subjects estimated by ANOVA model 1 (correction for age and gender). Only effects significant in cohort 1, subpopulation 2 (A+T+N+ AD vs A-T-N- SCD patients) and validation cohort 2 are shown, in concordance with figure 1. Abbreviations: imaginary coherence (iCoh), phase lag index (PLI), weighted phase lag index (wPLI), amplitude envelope correlation (AEC), corrected amplitude envelope correlation (AEC-c), coherence (Coh), phase locking value (PLV), standard deviation (SD).*

| **FC measure** | **SCD**  **(Mean, SD )** | **AD**  **(Mean, SD)** | **F-statistic** | **Corrected**  **p-value** | **Effect size (Cohen’s d)** |
| --- | --- | --- | --- | --- | --- |
| ***Theta (4-8Hz)*** |  |  |  |  |  |
| iCoh | 0.054±0.011 | 0.059±0.011 | 28.28 | 8.70*10^-7^ | 0.53 |
| PLI | 0.142±0.039 | 0.158±0.041 | 34.82 | 3.81*10^-8^ | 0.59 |
| wPLI | 0.100±0.026 | 0.111±0.028 | 36.64 | 1.62*10^-8^ | 0.60 |
|  |  |  |  |  |  |
| ***Alpha (8-13Hz)*** |  |  |  |  |  |
| AEC-c | 0.546±0.035 | 0.530±0.023 | 28.98 | 6.20*10^-7^ | -0.53 |
| AEC | 0.661±0.038 | 0.629±0.028 | 81.06 | 4.26*10^-17^ | -0.90 |
| Coh | 0.540±0.040 | 0.514±0.027 | 49.32 | 4.59*10^-11^ | -0.70 |
| PLV | 0.438±0.088 | 0.394±0.067 | 26.62 | 1.94*10^-6^ | -0.51 |
|  |  |  |  |  |  |
| ***Beta (13-30Hz)*** |  |  |  |  |  |
| AEC-c | 0.529±0.014 | 0.523±0.011 | 13.81 | 5.78*10^-4^ | -0.37 |
| AEC | 0.596±0.021 | 0.587±0.019 | 13.68 | 6.18*10^-4^ | -0.37 |
| Coh | 0.473±0.017 | 0.466±0.017 | 7.22 | 0.020 | -0.27 |

| **FC measure** | **SCD**  **(Mean, SD )** | **AD**  **(Mean,SD)** | **F-statistic** | **Corrected**  **p-value** | **Effect size (Cohen’s d)** |
| --- | --- | --- | --- | --- | --- |
| ***Theta (4-8Hz)*** |  |  |  |  |  |
| Coh | 0.512±0.024 | 0.514±0.20 | 33.50 | 7.15*10^-8^ | -0.57 |
| PLV | 0.369±0.047 | 0.380±0.041 | 39.14 | 5.00*10^-9^ | -0.62 |
|  |  |  |  |  |  |
| ***Alpha (8-13Hz)*** |  |  |  |  |  |
| AEC-c | 0.546±0.035 | 0.530±0.023 | 32.89 | 9.52*10^-8^ | -0.57 |
| AEC | 0.661±0.038 | 0.629±0.028 | 32.17 | 1.34*10^-7^ | -0.56 |
|  |  |  |  |  |  |
| ***Beta (13-30Hz)*** |  |  |  |  |  |
| AEC-c | 0.529±0.014 | 0.523±0.011 | 17.61 | 8.25*10^-5^ | -0.42 |
| AEC | 0.596±0.021 | 0.587±0.019 | 13.22 | 7.80*10^-4^ | -0.36 |

*Supplementary table 2: Difference in functional connectivity (FC) between SCD and AD subjects estimated by ANOVA model 2 (correction for age, gender and global relative power). Only effects significant in cohort 1, subpopulation 2 (A+T+N+ AD vs A-T-N- SCD patients) and validation cohort 2 are shown, in concordance with figure 2. Abbreviations: imaginary coherence (iCoh), phase lag index (PLI), weighted phase lag index (wPLI), amplitude envelope correlation (AEC), corrected amplitude envelope correlation (AEC-c), coherence (Coh), phase locking value (PLV), standard deviation (SD).*

*Supplementary figure 1: Topographical distribution of the median difference in Z-score of the PLI and AEC-c between the SCD and AD subjects. Results for the PLI and AEC-c in each bandwidth are shown. Blue indicates lower values in the AD group and red indicates lower values in de SCD group.*

*Supplementary figure 2A & 2B: Summary of observed differences in ANOVA model 1, shown as effect size (Cohen’s d), between AD and SCD subjects in different regions for the AEC-c and PLI in each bandwidth. The values of each channel and each subject were averaged into 4 regional clusters frontal (channels Fp1, Fp2, F3, F4, F6, F8, F7), temporal (channel T3, T4, T5, T6), central (channel C3, C4, Cz) and parieto-occipital (P3, P4, Pz, O1, O2).* *The significant effect sizes of the comparisons made in the entire cohort 1 (n=411) are shown. Effects that could not be reproduced in the subset populations or cohort 2 were left out. Red blocks represent a higher and blue blocks a lower level of functional connectivity in AD subjects compared to SCD subjects. The size of the blocks and the number shown in the blocks represent the size of the effect.*

*Supplementary figure 3A-C: Correlation coefficients (r) between functional connectivity measures in the theta (A), alpha (B) and beta (C) bandwidths. In each figure, the coefficients on the right are corrected for changes in relative power and the coefficients on the left are the uncorrected values.*
