# Supplementary figures and images for: Reproducibility of EEG functional connectivity in Alzheimer’s disease
Source: Alzheimers Res Ther. 2020 Jun 3;12:68. doi: 10.1186/s13195-020-00632-3 (PMC7271479; doi:10.1186/s13195-020-00632-3)

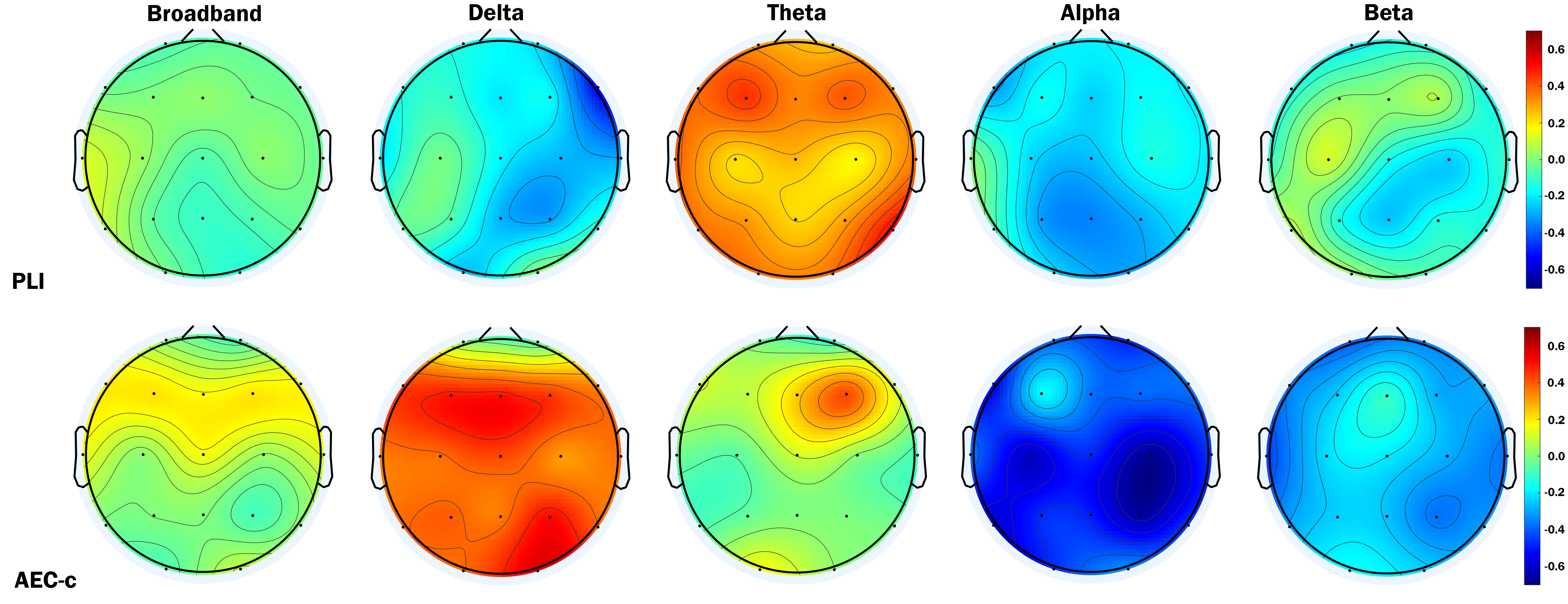

Supplement: Supplementary file 1 — Additional file 1: Supplementary Table 1. Difference in functional connectivity (FC) between SCD and AD subjects estimated by ANOVA model 1 (correction for age and gender). Supplementary Table 2. Difference in functional connectivity (FC) between SCD and AD subjects estimated by ANOVA model 2 (correction for age, gender and global relative power). Supplementary Figure S1. Topographical distribution of the median difference in Z-score of the PLI and AEC-c between the SCD and AD subjects. Supplementary Figure S2A & 2B. Summary of observed differences in ANOVA model 1, shown as effect size (Cohen’s d), between AD and SCD subjects in different regions for the AEC-c and PLI in each bandwidth. Supplementary Figure S3A-C. Correlation coefficients (r) between functional connectivity measures in the theta (A), alpha (B) and beta (C) bandwidths. In each figure, the coefficients on the right are corrected for changes in relative power and the coefficients on the left are the uncorrected values. [file 13195_2020_632_MOESM1_ESM.zip › supplementary figure 1.tif]

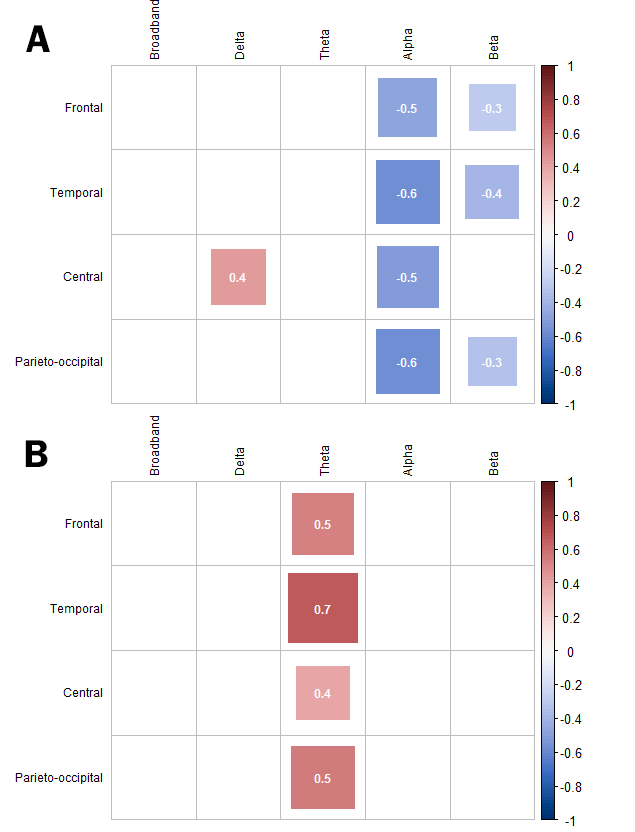

Supplement: Supplementary file 1 — Additional file 1: Supplementary Table 1. Difference in functional connectivity (FC) between SCD and AD subjects estimated by ANOVA model 1 (correction for age and gender). Supplementary Table 2. Difference in functional connectivity (FC) between SCD and AD subjects estimated by ANOVA model 2 (correction for age, gender and global relative power). Supplementary Figure S1. Topographical distribution of the median difference in Z-score of the PLI and AEC-c between the SCD and AD subjects. Supplementary Figure S2A & 2B. Summary of observed differences in ANOVA model 1, shown as effect size (Cohen’s d), between AD and SCD subjects in different regions for the AEC-c and PLI in each bandwidth. Supplementary Figure S3A-C. Correlation coefficients (r) between functional connectivity measures in the theta (A), alpha (B) and beta (C) bandwidths. In each figure, the coefficients on the right are corrected for changes in relative power and the coefficients on the left are the uncorrected values. [file 13195_2020_632_MOESM1_ESM.zip › supplementary figure 2.tif]

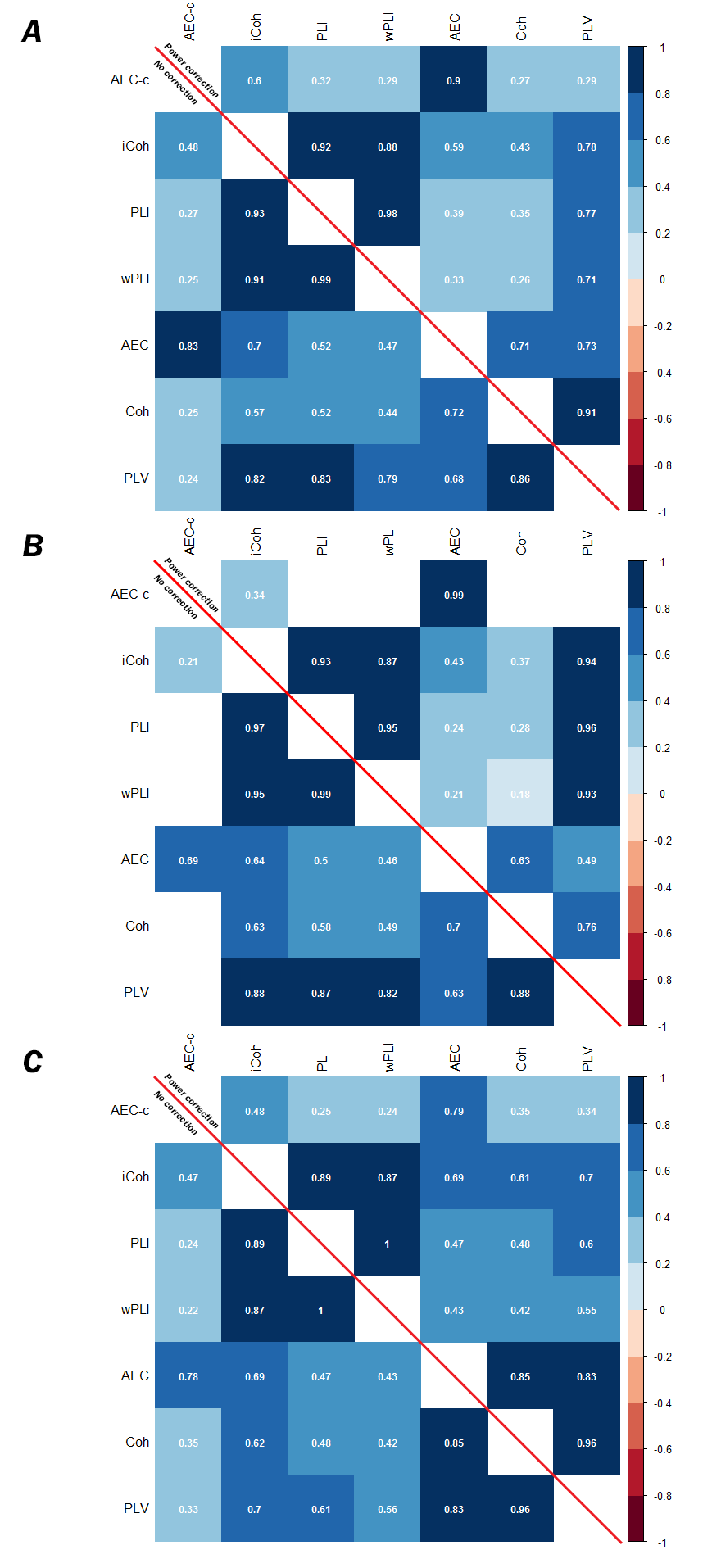

Supplement: Supplementary file 1 — Additional file 1: Supplementary Table 1. Difference in functional connectivity (FC) between SCD and AD subjects estimated by ANOVA model 1 (correction for age and gender). Supplementary Table 2. Difference in functional connectivity (FC) between SCD and AD subjects estimated by ANOVA model 2 (correction for age, gender and global relative power). Supplementary Figure S1. Topographical distribution of the median difference in Z-score of the PLI and AEC-c between the SCD and AD subjects. Supplementary Figure S2A & 2B. Summary of observed differences in ANOVA model 1, shown as effect size (Cohen’s d), between AD and SCD subjects in different regions for the AEC-c and PLI in each bandwidth. Supplementary Figure S3A-C. Correlation coefficients (r) between functional connectivity measures in the theta (A), alpha (B) and beta (C) bandwidths. In each figure, the coefficients on the right are corrected for changes in relative power and the coefficients on the left are the uncorrected values. [file 13195_2020_632_MOESM1_ESM.zip › supplementary figure 3.tif]
